# MoG+3.0: expanded structural variant visualization and integration of genomic data from five newly analyzed mouse strains

**DOI:** 10.1007/s00335-025-10168-2

**Published:** 2025-11-19

**Authors:** Toyoyuki Takada, Hideyuki Miyazawa, Masanobu Yamagata, Masaru Tamura, Atsushi Yoshiki, Atsushi Toyoda, Hideki Noguchi, Hiroshi Masuya

**Affiliations:** 1 Integrated Bioresource Information Division, RIKEN BioResource Research Center, 3-1-1 Koyadai, Tsukuba, Ibaraki 305-0074 Japan; 2 Center for Genome Informatics, Joint Support-Center for Data Science Research, Research Organization of Information and Systems, 1111 Yata, Mishima, 411-8540 Japan; 3 Mouse Phenomics Division, RIKEN BioResource Research Center, 3-1-1 Koyadai, Tsukuba, Ibaraki 305-0074 Japan; 4 Experimental Animal Division, RIKEN BioResource Research Center, 3-1-1 Koyadai, Tsukuba, Ibaraki 305-0074 Japan; 5 Comparative Genomics Laboratory, National Institute of Genetics, 1111 Yata, Mishima, 411-8540 Japan; 6 Advanced Genomics Center, National Institute of Genetics, 1111 Yata, Mishima, 411-8540 Japan; 7 Present Address: Department of Anatomy and Structural Biology, Graduate School of Medicine, University of Yamanashi, 1110 Shimokato, Chuo, Yamanashi 409-3898 Japan

**Keywords:** Mouse, Wild-derived inbred strains, Structural variation, Database

## Abstract

The MoG+ (Mouse Genome database with high added value), which has been operational since 2019, provides detailed visualization of genomic variations in mouse experimental strains, including wild-derived inbred strains, in particular those maintained at RIKEN BioResource Research Center. Here, we report on the enhancement of MoG+ by inclusion of the latest genome reports and by incorporation of structural variation (SV) information from recent analyses. The latter included long-read sequencing studies of the disease model strains FLS/Shi, NC/Nga, STR/OrtCrlj, JF1/Ms, and MSM/Ms. These studies described SNPs (4,482,628 to 19,644,769), short indels (726,646 to 2,391,782), and SVs such as insertions (32,949 to 131,311), deletions (28,259 to 102,226), and inversions (32 to 164). The new version of the database, which is named MoG+3.0, includes a feature that allows users to visually observe variants in the five strains. Through enhancement of the functionality of the database, SVs have been incorporated and visualized, allowing users to visually examine variants that were difficult to detect using only short-read-based resequencing data. The inclusion of the new variant data, along with enhanced features such as visualization, is expected to serve as a valuable resource for studies of disease and phenotype in experimental mice.

**Supplementary Information:**

The online version contains supplementary material available at 10.1007/s00335-025-10168-2.

## Introduction

Genome databases are fundamental to modern biomedical research. Widely used genome browsers such as the Ensembl Genome Browser (Harrison et al. 2024), NCBI Genome Data Viewer (Rangwala et al. 2024), and UCSC Genome Browser (Perez et al. 2025) have been developed and maintained by major research institutions to support diverse life science disciplines. These platforms are essential tools for genetic, developmental biology, neuroscience, and human disease studies, particularly those utilizing model organisms such as the laboratory mouse, including both classical inbred strains and genetically diverse wild-derived strains.

The laboratory mouse plays a pivotal role in experimental studies due to its genetic and physiological similarities to humans, the rich collection of well-characterized inbred strains, and its compatibility with a wide array of experimental techniques. The genomes of widely used inbred mouse strains are mosaics derived from at least three divergent subspecies: *Mus musculus domesticus*, *M. m. musculus*, and *M. m. castaneus* (Yang et al. 2011; Keane et al. 2011). In addition, *M. m. molossinus*, a hybrid of *musculus* and *castaneus*, also contributes to the genomic makeup of several strains (Yonekawa et al. 1988; Takada et al. 2013). These evolutionary layers highlight the complexity of mouse genomic resources in biomedical applications.

To effectively exploit the full potential of mouse genomic resources, comprehensive and detailed data on genomic variations are essential. High-resolution genome variation data, such as single nucleotide variants (SNVs), insertions/deletions (indels), and structural variants (SVs) of 50 bp or more, are critical for interpreting phenotypic differences among mouse strains.

The publication of reference genomes and comparative resequencing datasets has advanced our understanding of genetic diversity in laboratory mice (Keane et al. 2011; Lilue et al. 2018) and has aided the design of experiments using genome editing and trait mapping studies. Wild-derived inbred strains are a particularly valuable genetic resource for the exploration of the origins and diversity of laboratory mouse genomes. For example, the “Mishima Battery” strains established by Prof. Kazuo Moriwaki and colleagues represents a key contribution to this field (Moriwaki 1994; Takada et al. 2022). The Mishima Battery is a unique panel of ten strains derived from geographically distinct wild populations, including representatives of *domesticus*, *musculus*, *castaneus*, and *molossinus* subspecies (Takada et al. 2022). It is thought that *molossinus* originated from hybridization between *musculus* and *castaneus* (Yonekawa et al. 1988), although most of its genome is derived from *musculus* (Yonekawa et al. 1988; Bonhomme et al. 1989). These strains include a broad spectrum of natural variation and have been maintained with complete pedigree records. The phenotypic diversity for behavioral and physiological traits in these strains makes them invaluable for dissecting genotype-phenotype relationships (Takahashi et al. 2006; Takada et al. 2022).

Genomic information on these strains is publicly available through the MoG+ database (Mouse Genome Database with High Added Value; https://molossinus.brc.riken.jp/mogplus/) that was developed and maintained by our group (Takada et al. 2022). MoG+ was initially developed as a genome variation database to comprehensively catalog and publicly provide SNVs and short-indel variations across the ten wild-derived inbred mouse strains of the Mishima Battery. By enabling visualization of subspecies-specific variants and linking them to phenotypic data, MoG+ provides a valuable resource for exploring genotype-phenotype relationships. The MoG+ database provides direct links to supporting datasets, publications (Takada et al. 2015, 2022), and downloadable resources.

Recent advances in sequencing technologies such as long-read platforms have enabled precise characterization of complex SVs (Roberts et al. 2013; Jain et al. 2016; Sedlazeck et al. 2018), further refining our capacity to understand genome function. The public release of SV datasets (e.g., Ferraj et al. 2023; Arslan et al. 2023) has enabled researchers to incorporate SVs into genetic analyses, thereby improving the interpretability of functional genomics. In response to an increasing demand for centralized and accessible genome variation data, we have now developed MoG+ version 3.0 (MoG+3.0). In this version, genome-wide exploration of SVs in disease studies is now possible for the mouse strains FLS/Shi, NC/Nga, STR/OrtCrlj, JF1/Ms, and MSM/Ms, in addition to the SNV and amino acid substitution analyses. The FLS/Shi mouse strain spontaneously develops fatty liver without obesity and non-alcoholic steatohepatitis (NASH), making it a valuable model for studying obesity-independent fatty liver progression and chronic liver inflammation (Soga et al. 1999, 2010). The NC/Nga strain exhibits atopic dermatitis (AD)-like skin lesions with Th2-skewed immune responses under conventional housing conditions, making it an ideal model for allergy and skin inflammation research (Suto et al. 1999). The STR/OrtCrlj mouse naturally develops osteoarthritis with age, resembling human OA through progressive cartilage degeneration and joint remodeling (Watters et al. 2007). Among wild-derived strains, MSM/Ms, a *M. m. molossinus* inbred line, is widely used for SNP discovery (Takada et al. 2013) and allele-specific gene expression studies (Kondo et al. 2019); this strain offers the possibility of crucial insights into intersubspecific genomic variations that are not captured in classical laboratory strains (Takada et al. 2022). JF1/Ms, also derived from *M. m. molossinus*, has a high probability of being the ancestral genomic contributor to classical laboratory strains (Takada et al. 2013) and is crucial in exploring subspecies diversity and phenotype-associated genetic differences (Takada et al. 2022).

The functionality of MoG+3.0 has been expanded by the addition of genome-wide SV visualization. In combination with strain-specific information from disease models and genomic diversity data from strains with different genetic backgrounds, MoG+3.0 offers a powerful tool for experimental design and interpretation of mouse-based biomedical research.

### Data acquisition of new genome sequencing data by hybrid-assembly

Long-read and short-read sequencing have been performed using five RIKEN BRC mouse strains: FLS/Shi, NC/Nga, STR/OrtCrlj, JF1/Ms, and MSM/Ms (Table S1). Details of sequencing platforms, read lengths, and sequencing depths for both long-read and short-read sequencing are provided in Table S2. In brief, long-read sequencing using the Sequel II platform yielded average read lengths ranging from 17,326 bp (MSM/Ms) to 21,218 bp (FLS/Shi), with sequencing depths between x86 (JF1/Ms) and x135 (STR/OrtCrlj). Short-read sequencing using the Illumina HiSeq2500 and NovaSeq6000 platforms produced read lengths of approximately 248–250 bp, with depths ranging from x68 (FLS/Shi) to x102 (JF1/Ms). For both sequencing approaches, high-molecular weight genomic DNAs for the JF1/Ms and MSM/Ms strains were obtained from frozen samples of the same individuals used in a previous study (Takada et al. 2013), ensuring consistency across datasets. The combination of high-quality long-read and short-read data enables robust SV detection and comprehensive genome analysis.

### Variant detection

Variant analysis was conducted by comparing the genomes of the mouse strains FLS/Shi, NC/Nga, STR/OrtCrlj, JF1/Ms, and MSM/Ms against the C57BL/6J reference genome (GRCm39/mm39). The results are summarized in Table 1. The number of single nucleotide polymorphisms (SNPs) ranged from 4.5 to 19.6 million across strains. In addition, the number of short insertions and deletions (short indels, ≤ 50 bp) ranged from 1.45 to 4.73 million, with 0.73 to 2.33 million insertions and 0.72 to 2.39 million deletions. SVs (≥ 50 bp) were also detected in substantial numbers, with a range of 61,208 to 233,537 among strains. The variants included insertions (32,949–131,311), deletions (28,259–102,226), and inversions (32–164). The highest variant counts were observed in the wild-derived strains JF1/Ms and MSM/Ms, reflecting their greater genetic divergence from the reference genome. This variant landscape highlights the extensive genomic diversity captured in MoG+3.0 and underscores the utility of incorporating both short- and long-read sequencing approaches for accurate detection of complex genetic variation.

**Table 1:** Summary of the genomic variations in each strain

	SNP	Short-InDel		SV		INV
		Insertion	Deletion	Insertion	Deletion	
FLS/Shi	5,136,880	811,578	813,887	36,804	31,737	44 (Min: 606, Max: 147,954)
NC/Nga	5,106,871	798,307	801,835	36,927	31,341	32 (Min: 1029, Max: 456,178)
STR/OrtCrlj	4,482,628	726,646	728,268	32,949	28,259	46 (Min: 247, Max: 375,873)
JF1/Ms	19,297,122	2,280,845	2,352,211	126,546	101,785	154 (Min: 165, Max: 442,023)
MSM/Ms	19,644,769	2,334,816	2,391,782	131,311	102,226	164 (Min: 138, Max: 622,211)

### Functional annotation of variants

Genome annotation with SnpEff (5.2c) classified the detected variants into four standard impact categories and mapped them to genomic features (Table S3). Among the five RIKEN strains, MODIFIER variants were predominated, ranging from 15.4 × 10^6^ to 65.2 × 10^6^ events and accounting for ~ 99.4% of all annotated sites per strain. Variants of LOW impact ranges from 64 k to 269 k, whereas MODERATE and HIGH impact variants were comparatively infrequent: 22.9 k to 89.5 k and 4.5 k to 12.5 k, respectively. Although relatively infrequent (< 0.04% of total calls), HIGH-impact variants represent potentially disruptive changes and were most abundant in the wild-derived strains JF1/Ms and MSM/Ms. Intronic substitutions constituted the largest positional class, with a range of 8.7 × 10^6^ to 38.1 × 10^6^ among strains; intergenic variants (3.3 × 10^6^–13.1 × 10^6^) were the next most common type. Downstream and upstream gene-proximal changes were detected at 1.6 × 10^6^–6.4 × 10^6^ and 1.6 × 10^6^–6.3 × 10^6^, respectively, while non-coding-transcript exon variants comprised 0.17 × 10^6^–0.75 × 10^6^ calls. This distribution is consistent with the predominance of non-coding regions in mammalian genomes and highlights reservoirs of regulatory variation.

### Repeat features

In the analysis of SVs, we examined the types of repeat elements present within each SV by applying a repeat-detection program to the insertions, deletions, and inversions identified through SV detection tools. As shown in Table S4, the results revealed distinct patterns among strains: MSM/Ms and JF1/Ms showed predominantly LINE elements; NC/Nga and STR/OrtCrlj exhibited a high proportion of ERV elements; and FLS/Shi displayed an intermediate composition, containing a relatively high proportion of both SINEs and ERVs.

### MoG+ database: structure and version overview

**Fig. 1:**
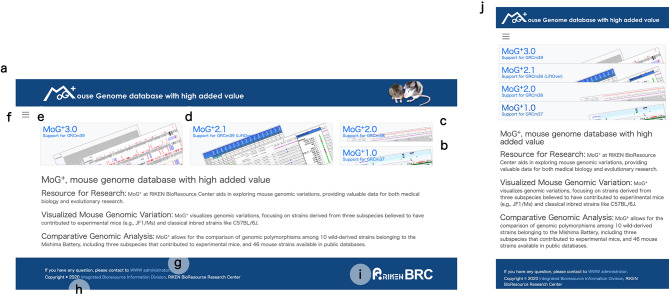
The structure of the MoG+ database is explained using the MoG+ top page. The MoG+ top page functions as a portal (**a**), featuring clickable banners that serve as entry points to each version of MoG+. These versions were independently constructed using different sets of mouse strains and using comparisons with different versions of reference genomes. Each version is named according to its corresponding reference genome. MoG+1.0 (**b**) includes genome realignment data for the wild-derived strains MSM/Ms and JF1/Ms (Takada et al. 2015). These data were originally mapped to GRCm37 and later lifted over to GRCm38. MoG+2.0 (**c**) contains data for 10 wild-derived strains from the Mishima Battery (Takada et al. 2022), directly mapped to GRCm38. MoG+2.1 (**d**) is an updated version that has been lifted over to the latest reference genome, GRCm39, ensuring compatibility with current genome resources. MoG+3.0 (**e**) includes information obtained through long-read sequencing of the five disease model strains in this report, including two from the Mishima Battery, and corresponds to GRCm39. This versioned structure enables users to flexibly access strain-specific genomic data based on the corresponding reference builds, allowing for highly reproducible and precise comparative analyses. In addition, the menu contains links to each version as well as to resources such as the data download page (**f**). Other features include a contact link for administrators (**g**), a link to the RIKEN BRC Integrated Information Development Office managing the database (**h**), and a link to RIKEN BRC itself (**i**). MoG+ is also compatible with multiple devices (**j**)

The MoG+ portal has been improved in order to respond to the growing need for a centralized, user-friendly repository of mouse genome-variation data (Fig. 1). The current interface presents a set of linked banners that serve as entry points to multiple previously released versions of MoG+. These versions are maintained independently, as described below, were built using specific mouse strains and aligned against reference genome assemblies available at the time. Each version is named with respect to its reference genome: MoG+1.0 (formerly known as NIG_MoG) comprises genome resequencing data from the wild-derived MSM/Ms and JF1/Ms strains (Takada et al. 2015), originally aligned to GRCm37 and subsequently lifted over to GRCm38. MoG+2.0 contains data from ten wild-derived Mishima Battery strains (Takada et al. 2022), mapped directly to GRCm38. MoG+2.1 is an updated version of 2.0, lifted over to the latest reference genome GRCm39 to ensure compatibility with current genomic resources. This versioned structure provides users with flexible access to strain-specific genomic data aligned to the appropriate reference builds, supporting reproducible and accurate comparative analysis.

### Update to version 3.0 of MoG+: expanded structural variant support and enhanced visualization tools

**Fig. 2:**
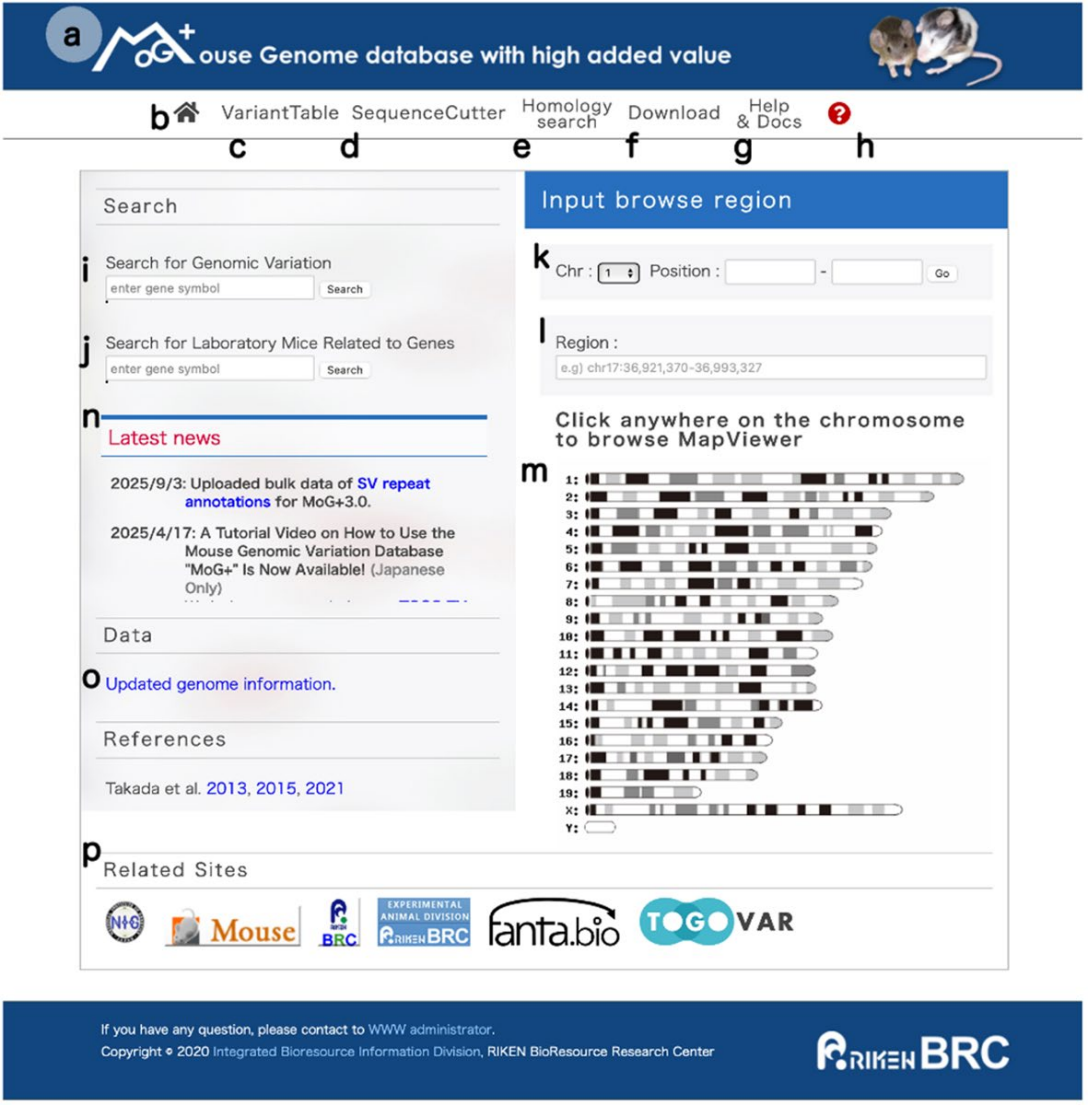
The MoG+3.0 top page. **a** Link to the MoG+ portal. **b** [Home Button] Returns to the MoG+3.0 top page. **c** [VariantTable] Enables searches of five disease model strains, including MSM/Ms and JF1/Ms from the Mishima Battery, the reference genome (C57BL/6J), and 52 inbred strains registered in the MGP REL2021 (https://www.ebi.ac.uk/eva/?eva-study=PRJEB53906). **d** [SequenceCutter] Currently supports only the GRCm38 reference genome in MoG+3.0. **e** [Homology Search] Performs homology searches based on GRCm38 coordinates; only available for the reference genome. **f** [Download] Allows downloading of various information related to the database. **g** [Help & Docs] Provides information on the use of data stored in MoG+. **h** Link to tutorial (same as for MoG+2.1). Some features of MoG+3.0 are also explained on TOGO TV (https://togotv.dbcls.jp/20250415.html; available in Japanese only). **i** [Gene Search Box] A search box that allows users to retrieve polymorphism information for a given gene by entering its gene symbol. **j** [Mouse Strain Search Box] Allows searching for disease model strains that are available from RIKEN BRC, including mutant and genetically engineered mice related to gene symbols. **k** [Search Box for Chromosome and Position Range: Direct Input] Enables data searches by specifying chromosome number and start/end positions of the display region. **l** [Search Box for Chromosome and Position Range: BED Format Input] Enables data searches by specifying display regions using BED format. **m** [Link to Chromosome Map Display Window for Arbitrary Position Selection] Allows users to select any chromosome position to view data for a desired region. **n** Function for providing various types of information. **o** Information on funds and support received for data production. **p** [Database-Related Websites] Links to other pages related to MoG+

**Fig. 3:**
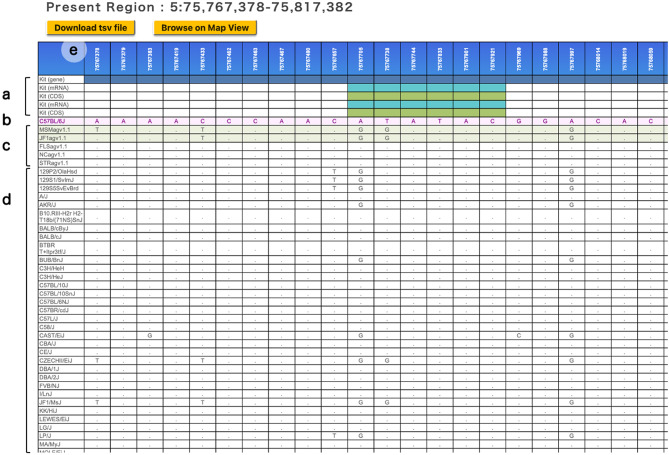
VariantTable (Fig. 2c) allows users to search five disease model strains analyzed in this study, including MSM/Ms and JF1/Ms from the Mishima battery, the reference genome, and 52 inbred strains registered in the MGP REL2021 dataset. Searches can be performed by genomic coordinates or gene symbols, and regions up to 300 kb can be displayed. The search results show nucleotide variants in each strain: SNVs with the reference are shown as the corresponding bases, while matches are indicated with a dot (.). VariantTable can be used to narrow down genomic regions potentially associated with phenotypes or disease susceptibility, and to assist in the design of various tools for genome editing. The results can be downloaded in CSV format, and users can jump to the corresponding region in the genome browser from a specified SNV coordinate, making it a highly intuitive and useful tool for comparative analysis. The figure shows an example of search results for the *Kit* gene region (chromosome 5: 75,767,378–75,817,382). Colored blocks in the coordinate track indicate genomic features: gene regions (dark blue), mRNA (light blue), and CDS (moss green) (**a**). **b** displays the reference bases from B6. Results from the five newly analyzed strains are shown. MSM/Ms and JF1/Ms, which are derived from the *molossinus* subspecies, are marked with light green (**c**). **d** shows variant information from public datasets

**Fig. 4:**
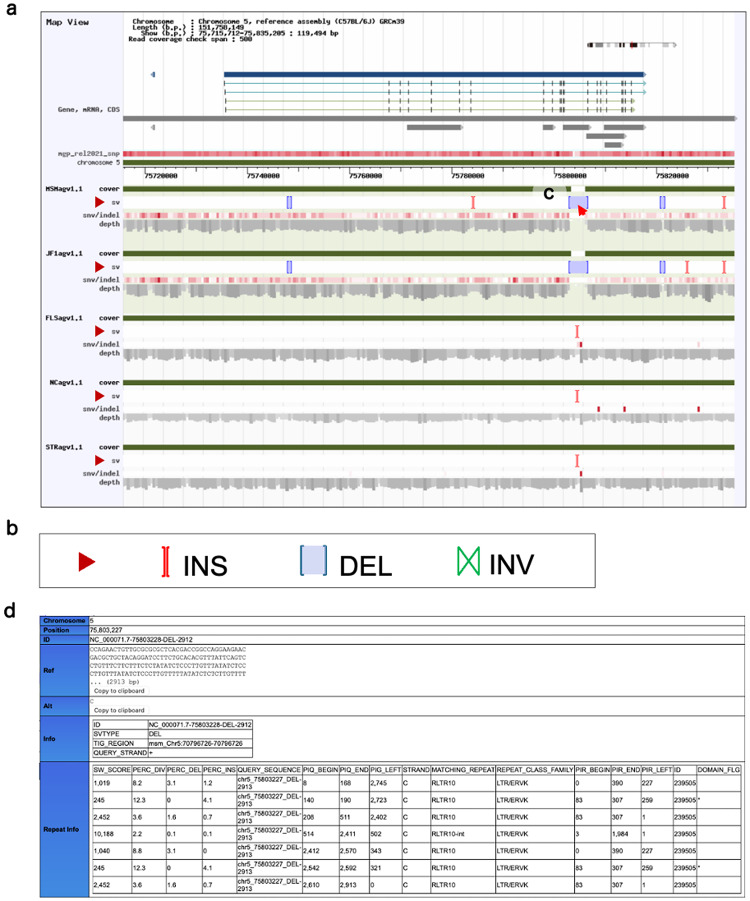
Newly implemented features include SV visualization and repeat information pop-ups. In the medium-range map view of the gene search function in MoG+3.0, structural variants (SVs) can now be displayed for the five strains analyzed in this study. The example shown depicts a region around the *Kit* gene region (Fig. 3), extended 20 kb upstream and downstream (**a**). SVs are identified by comparison with the reference genome C57BL/6J. Insertions are shown in green, deletions in red, and inversions in blue (**b**). By hovering the cursor over a specific SV graphic (**c**), detailed information such as its genomic coordinates and repeat type is displayed in a pop-up window (**d**)

**Fig. 5:**
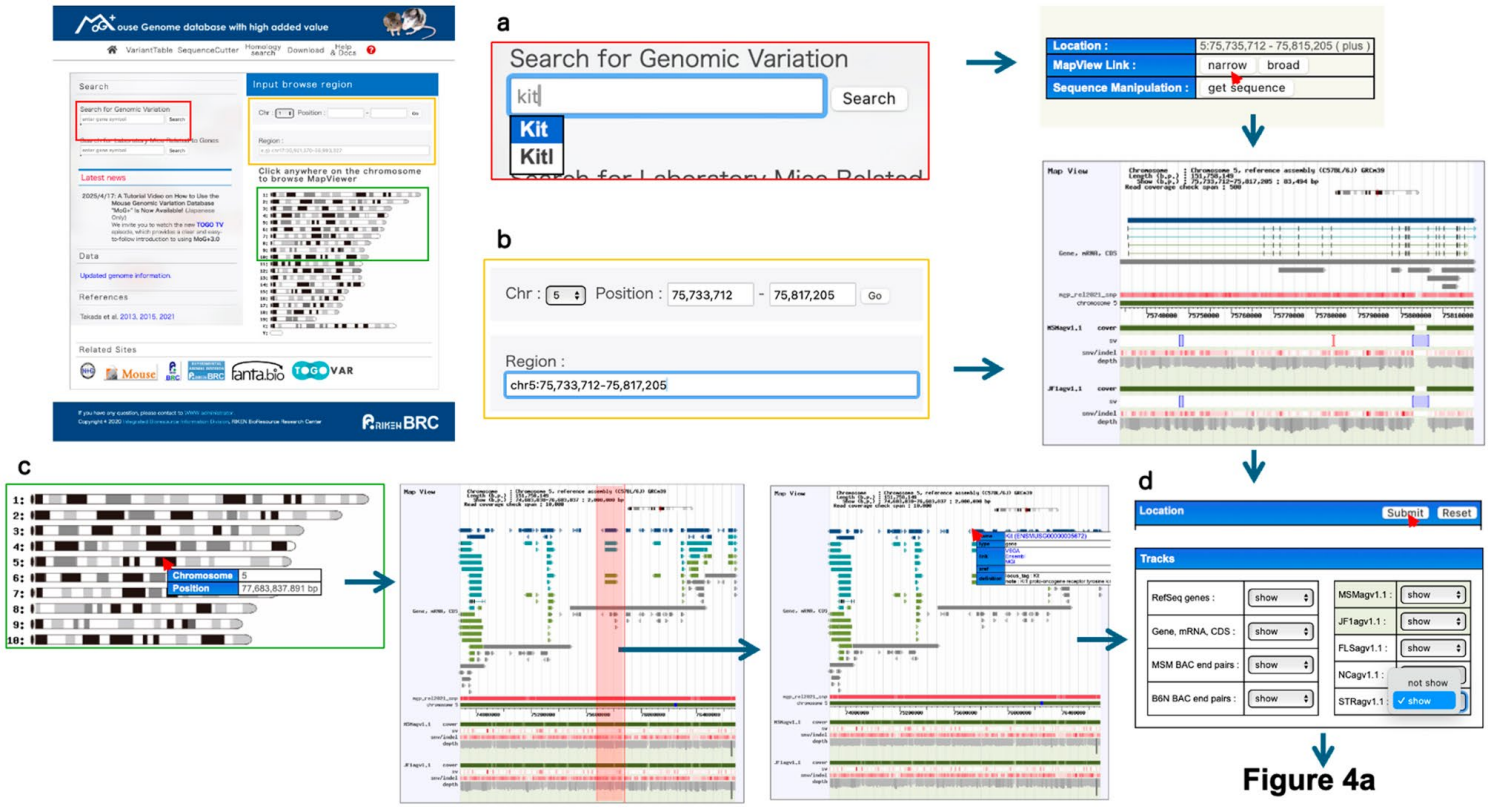
Procedure for accessing the genomic coordinates (chr5:75735712–75815205, BED format) corresponding to the Kit gene shown in Fig. 4. Three access routes (**a–c**) are available on the MoG+3.0 top page: **a** via gene-symbol search—typing a symbol triggers suggestions; selecting one opens the gene page, and the “Map” link displays its genomic range; **b** by directly entering coordinates (BED format) into the coordinate box or upper display box to show the desired region; **c** by clicking on the pseudo-karyotype to view nearby sequences, then defining upstream and downstream points to display that range; **d** in the medium-scale maps obtained by these methods, the MSM/Ms and JF1/Ms strains are displayed on the initial screen. By selecting a strain to display and clicking “Submit,” the information for the selected strain is shown

MoG+ version 3.0 (Fig. 2) seamlessly integrates and visualizes comprehensive genomic-diversity datasets spanning multiple laboratory strains, including two from the Mishima Battery, and thereby broadens the database’s relevance to evolutionary, functional, and disease-modeling research. The new version of the database includes intuitive analytical tools for interrogating SNVs and amino-acid substitutions and for the first time enables genome-wide exploration of SVs in five key strains: FLS/Shi, NC/Nga, STR/OrtCrlj, JF1/Ms, and MSM/Ms. In addition, MoG+ includes the VariantTable feature (Fig. 3), which allows users to compare public SNV data with those from strains we have analyzed. In MoG+3.0, this feature has been upgraded by referencing REL2021 (PRJEB53906) to retrieve and incorporate appropriate datasets. Furthermore, the latest update includes newly obtained SV information based on long-read sequencing analyses. This SV data is now viewable on both the medium-scale map and the gene structure visualization pages (Fig. 4a). When zoomed in, users can also display the short-read alignment map alongside the resequencing map. Additionally, we have worked to integrate repeat element information for the detected SVs into the MoG+3.0 mouse genome database (Fig. 4b). As a result, users can now visually confirm repeat elements along with SVs directly in the browser (Fig. 4c). Figure 4d shows the types of SVs: INS indicates insertion, DEL indicates deletion, and INV indicates inversion. Figure 5 illustrates how a specific genomic region can be examined and how the search results shown in Fig. 4a were accessed from the top page of MoG+3.0, while Supplementary Fig. 1 presents an example of search results for another gene.

### Links to and collaboration with other databases

Each gene entry in MoG+ is enriched with links to a wide range of external biological databases, including Ensembl (Dyer et al. 2025), MGI (Baldarelli et al. 2024), GO (The Gene Ontology Consortium 2021), Reactome (Milacic et al. 2024), UniProt (UniProt Consortium 2021), RefSeq (O’Leary et al. 2016), and KEGG (Kanehisa et al. 2016). Gene entries also provide references to human orthologs and clinical variant resources such as OMIM (Amberger et al. 2015), MedGen (Louden 2020), and TogoVar (Mitsuhashi et al. 2022), facilitating comprehensive functional and translational interpretation of mouse genetic variation. Recent updates to the FANTOM web resource have expanded the annotation of long non-coding RNAs (lncRNAs) based on perturbation experiments in iPSCs and Hi-C analysis; this resource is now accessible through the intuitive ZENBU-Reports interface (Nobusada et al. 2025). In connection with these updates, the newly launched fanta.bio (https://fanta.bio) platform provides access to transcribed *cis*-regulatory elements (CREs) enriched with genetic and epigenetic metadata. Genome variation data across diverse mouse strains that include these CREs, are also provided by the MoG+ dataset, enabling comprehensive analysis of regulatory elements within the context of natural genetic diversity. To expand the translational utility of mouse genomic variation data, MoG+ is currently working toward connection with TogoVar, a comprehensive Japanese genetic variation database (Mitsuhashi et al. 2022). TogoVar provides allele frequency data derived from Japanese populations along with rich annotations on molecular consequence, pathogenicity, and supporting literature. By enabling cross-referencing between MoG+ and TogoVar entries, we aim to facilitate comparative analyses between mouse and human genomes, particularly in evolutionarily conserved or functionally relevant regions.

These upgrades will enhance the role of MoG+ as a resource both for model organism research and for bridging basic and clinical genomics. These advances demonstrate that the MoG+3.0 upgrades, most notably the incorporation of long-read assemblies, will fill a critical gap in the genomic analyses of multiple wild-derived and disease-model mice by transforming previously elusive SVs and repetitive sequence landscapes into directly explorable data. The interconnections with multiple versions of reference genome coordinates and the enrichments provided by referencing with Ensembl, OMIM, TogoVar, and the FANTOM/fanta.bio ecosystem, have enabled MoG+ to evolve into an integrated platform that bridges basic, translational, and comparative genomic research. This resource is expected to support the design of precise genome-editing experiments, refine genotype-to-phenotype mapping across laboratory strains available from RIKEN BRC, and, through planned connections with human variant resources, accelerate the bidirectional exchange of insights between mouse models and human disease genetics.

### Future perspectives

Here, we present an updated overview of genome information for wild-derived and disease-model inbred mouse strains from RIKEN BRC in Japan and describe the enhancements to the MoG+ database through the integration of long-read-based genome analysis. Notably, the samples analyzed in this study are identical to those used in prior research on JF1/Ms and MSM/Ms (Takada et al. 2013, 2022), eliminating inter-individual genomic variation and ensuring high-quality, consistent results. The adoption of long-read assemblies has enabled the direct identification of SVs that were previously undetectable using short-read sequencing. These SVs can now be visualized without the need for statistical inference, allowing researchers to detect variants that may affect not only exons but also introns and *cis*-regulatory regions. The accuracy of repeat sequence identification has also been significantly improved. Together, these advances provide high-resolution genomic data that support the design of precise genome-editing experiments and other functional studies. The current version of MoG+ supports multiple reference genome builds: GRCm39 for SVs, and GRCm37, GRCm38, and GRCm39 for SNV and indel annotations. Looking ahead, selected datasets also will be made accessible through platform such as fanta.bio, a database designed for integrative visualization of functional genome annotations, with a particular focus on CREs such as promoters and enhancers. Importantly, because less than about 6% of the *molossinus* genome has been introgressed into the genomes of commonly used classical inbred mouse strains such as C57BL/6 (Takada et al. 2013), future studies will be able to identify SVs specific to the haplotypes of these strains and utilize them as informative markers for phenotype analysis.

## Materials and methods

### Mice

We analyzed genomic DNAs from FLS/Shi, NC/Nga, STR/OrtCrlj, JF1/Ms, and MSM/Ms. The genomic DNAs of JF1/Ms and MSM/Ms mice were extracted in this study from cryopreserved specimens previously used in earlier studies (Takada et al. 2013). FLS/Shi (Strain ID; RBRC03707), NC/Nga (RBRC01059), STR/OrtCrlj(RBRC06803), JF1/Ms (RBRC00639), and MSM/Ms (RBRC00209) are maintained as pedigreed breeding stocks at the RIKEN BRC. Samples used in the sequencing study are listed in Table S1. All animal experiments were approved by the Animal Care and Use Committee of RIKEN BRC.

### Acquisition of new genome sequencing data by hybrid-assembly

Long-read genomic sequencing of five mouse strains for *de novo* assembly was performed using PacBio Sequel II SMRT Cell system. Short-read genomic sequencing for hybrid assembly was performed using HiSeq2500 for JF1/Ms (PE600) and NovaSeq6000 (Illumina) for FLS/Shi, MSM/Ms, NC/Nga, and STR/OrtCrlj (PE500) according to the manufacturer’s protocols. We assembled the genomes using Falcon v1.8.1 (Chin et al. 2016) and Wtdbg2 v2.5 (Ruan and Li 2020) to create two contig sets from their long-read datasets. Parameters for Wtdbg2 were ‘-x sequel’ and ‘-g 3G’, as estimated genome size, and those of Falcon were ‘genome_size = 2800000000’, ‘seed_coverage = 60, and ‘length_cutoff = 9000’. These two contig sets were individually improved by a first polish using the long-read data with Arrow (SMRT link version 2.3.3), then using short-read data with NextPolish (Hu et al. 2020), and finally haplotigs were eliminated using Purge_dups (Guan et al. 2020). The two contig sets were merged with Quickmerge (Chakraborty et al. 2016). The merged contigs were organized into chromosomal level sequences with RagTag (Alonge et al. 2022) using the mouse genome assembly GRCm39 as the reference. Mitochondrial genomes were independently assembled using Platanus (Kajitani et al. 2019) from short-read datasets, and then merged into this assembly, and finally the scaffolds were sorted by length. The completeness of final assemblies was evaluated by the benchmarking universal single-copy orthologs (BUSCO) v5.1.2 using the Glires data sets (glires_odb10) (Manni et al. 2021).

### Variant call

Filtering and trimming of short reads were executed with Fastp (Chen et al. 2018), mapping to the reference genome was conducted with BWA (Li and Durbin 2010), and the genomic variants including SNPs and short indels were identified with the Genome Analysis Toolkit (GATK) (https://gatk.broadinstitute.org/hc/en-us) using these short read data sets. Genomic variants and SVs were merged to create the VCF files, which were used in the database. SV visualization was carried out using data on genomic structural variations identified between the scaffolds and the reference using PAV (Ebert et al. 2021). The mouse genome assembly GRCm39 was used as the reference in these analyses. The merged VCF files (HybridVCF) were generated using small variants (less than 50 bp) data from the GATK outputs and large variants (equal and greater than 50 bp) data from the PAV outputs.

### Repeat masking

Repetitive sequences on the HybridVCF of search strain were identified with RepeatMasker v4.1.5 (Smit et al. 2013), with the option ‘-species “Mus musculus”’.

## Supplementary Information

Below is the link to the electronic supplementary material.

Supplementary Material 1

## Data Availability

The sequence data from this study have been submitted to the DDBJ Sequence Read Archive (DRA) under accession numbers DRA011245 (excluding JF1 short-read data) and DRA006630 (JF1 short-read data; only the paired-end reads were used in this study). All Supplemental Material is available on the download page of MoG+ (https://molossinus.brc.riken.jp/pub/MoGplus30data/). The analysis for acquisition of genomic data and the data analysis for the genomic variation detection were performed at NIG and ROIS-DS.
