# Supplementary material for: MoG+3.0: expanded structural variant visualization and integration of genomic data from five newly analyzed mouse strains
Source: Mamm Genome. 2025 Nov 19;37(1):4. doi: 10.1007/s00335-025-10168-2 (PMC12630176; doi:10.1007/s00335-025-10168-2)
Supplement: Supplementary file 1 — Supplementary Material 1 [file 335_2025_10168_MOESM1_ESM.pdf]

Takada et al. Supplementary Information

MoG+3.0: Expanded Structural Variant Visualization and Integration of Genomic Data from Five Newly Analyzed Mouse Strains

Table S1. Identities of the mice used in the sequencing study

Strain	ID	SEX	DOB	DOSC	F
FLS/Shi	201	F	2020/4/16	2020/7/3	(F131)+F16
NC/Nga	C02	F	2020/4/15	2020/7/3	(G unk.)+F11
STR/OrtCrlj	401	F	2020/4/15	2020/7/3	(G unk.)+F8G7
JF1/Ms*					
MSM/Ms*					

DOB: Date of birth

DOSC: Date of sample collection

F: The number of inbreeding generations the time used for the analysis

*: Published previously (Takada et al. 2013)

Table S2. Summary of sequencing readl engths and depths for long-read and short-read genomic analyses

	Long-reads (Sequel2)		Short-reads (HiSeq2500 [JF1 only], NovaSeq6000 [other 4 strains])	
Strain	Avg_len	Depth	Avg_len	Depth
FLS/Shi	21,218	x112	250	x68
NC/Nga	21,098	x121	250	x94
STR/OrtCrlj	21,098	x135	250	x93
JF1/Ms*	18,917	x86	248	x102
MSM/Ms*	17,326	x134	250	x90

* The high-molecular weight genomic DNA of both JF1 and MSM was purified from frozen samples of the same individual used for previous study (Takada et al. 2013).

Table S3. Functional impact classification of genomic variants by SnpEff program

Strain	HIGH Impact (%)	MODERATE Impact (%)	LOW Impact (%)	MODIFIER Impact (%)	Missense	Silent	Missense/Silent Ratio
FLS/Shi	0.056	0.159	0.424	99.361	27169	53229	0.5104
NC/Nga	0.035	0.167	0.438	99.359	29222	57042	0.5123
STR/OrtCrlj	0.04	0.148	0.414	99.398	22160	45721	0.4847
JF1/Ms	0.027	0.138	0.413	99.422	85665	190172	0.4505
MSM/Ms	0.027	0.136	0.411	99.426	86248	192779	0.4474

Table S4. The results of RepeatMasker analysis using SV sequences obtained from the PAV program

Category	FLS/Shi	NC/Nga	STR/OrtCrlj	JF1/Ms	MSM/Ms
SINEs	3.79	2.68	2.63	2.87	2.85
LINEs	41.57	45.21	46.54	52.92	53.08
LTR	25.79	27.95	27.64	23.75	23.47
DNA	0.21	0.11	0.11	0.11	0.12
Unclassified	0.45	0.35	0.22	0.34	0.21
Simple repeats	3.49	3.63	3.54	4.08	4.17
Low complexity	0.37	0.38	0.39	0.29	0.27
Satellites	0.42	0.34	0.34	0.31	0.35
Small RNA	0.04	0.03	0.04	0.03	0.04
Total interspersed	71.8	76.3	77.15	79.98	79.73

The numbers are expressed as percentages.

Supplementary_Figure 1

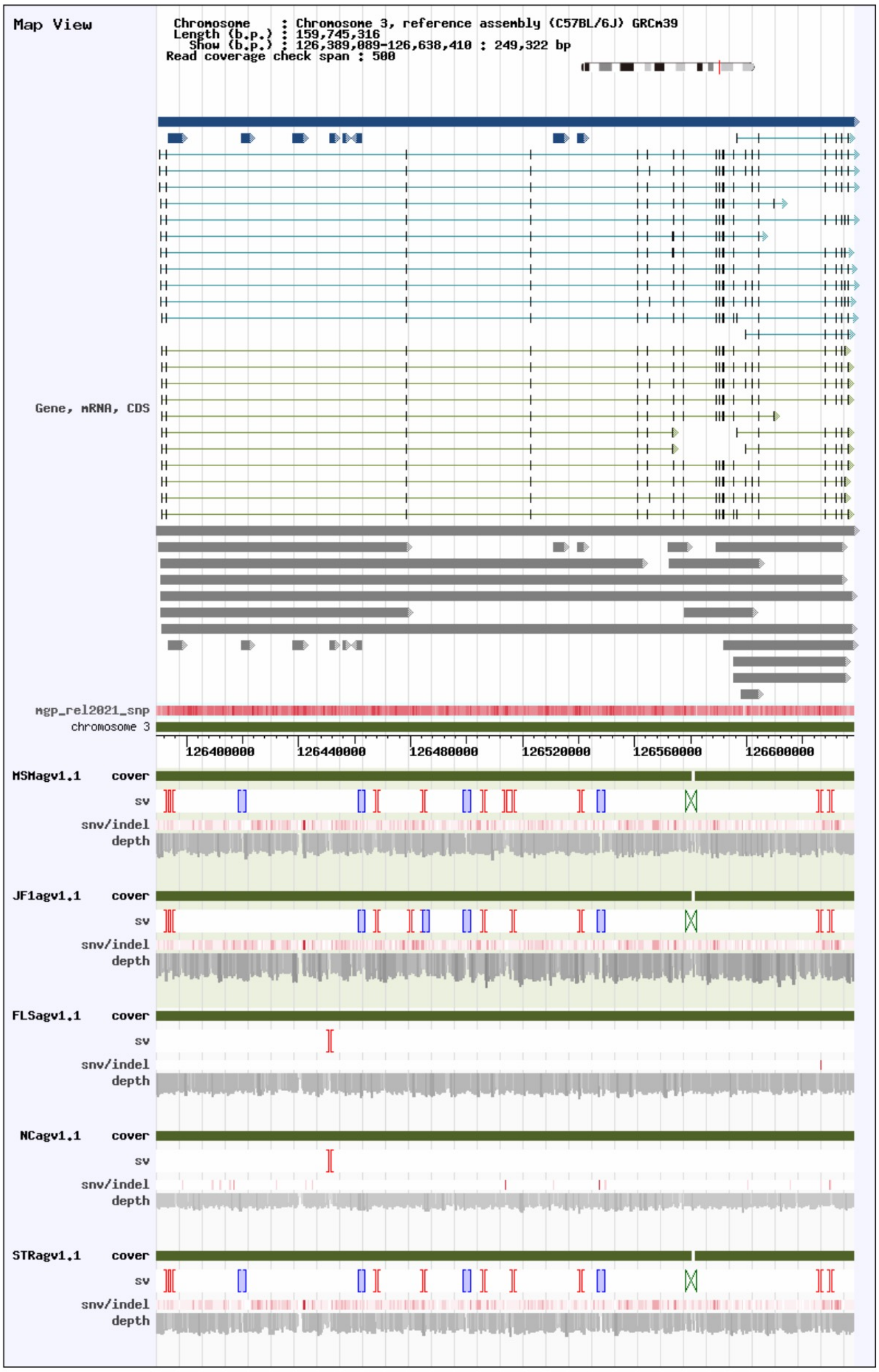

Figure legends

Supplementary Figure 1

Medium-scale map of Camk2d (calcium/calmodulin-dependent protein kinase II, delta) obtained as in Supplementary Figure 1. In MSM/Ms, JF1/Ms, and STR/OrtCrlj, insertions (INS), deletions (DEL), and inversions (INV) are observed.

Overview of the characteristics of the mouse strains featured in MoG+ 3.0

FLS/Shi

FLS/Shi (The Fatty Liver Shionogi) mouse is an inbred strain that spontaneously develops hepatic steatosis without accompanying obesity or diabetes, making it a unique and valuable model for studying liver diseases. In the original characterization, hepatocytes of neonatal FLS mice exhibited fine lipid droplets that enlarge with age, and liver triglyceride levels were five times higher than in control strains, despite normal serum lipid and lipoprotein profiles (Soga et al. 1999). To investigate the interaction between obesity and fatty liver progression, a congenic strain, FLS-$Lep^{ob/ob}$, was generated by introducing the Lep^{ob} mutation into the FLS background. This model displayed severe insulin resistance, advanced non-alcoholic steatohepatitis (NASH), and even progression to hepatocellular carcinoma, thereby providing a powerful system to study obesity-driven liver disease (Soga et al. 2010). Moreover, under NASH conditions, the FLS liver demonstrated locally increased expression of inflammation-associated molecules such as lipocalin-2, CXCL1, and CXCL9. These findings underscore the utility of FLS mice as a model that recapitulates chronic inflammatory responses seen in NASH (Semba et al. 2013).

NC/Nga

The NC/Nga mouse strain is widely recognized as a representative animal model for human atopic dermatitis (AD). When maintained under conventional housing conditions, these mice spontaneously develop eczematous skin lesions that closely resemble those observed in human AD patients. Histological analysis confirms the presence of key pathological features such as dermal infiltration by inflammatory cells and elevated serum IgE levels, making NC/Nga mice a valuable model for investigating AD pathogenesis and therapeutic approaches (Suto et al. 1999). Molecular studies have demonstrated the overproduction of Th2-specific chemokines, including the thymus- and activation-regulated chemokine TARC and macrophage-derived chemokine MDC, in lesional skin, implicating these mediators in the development of Th2-skewed immune responses in this model (Vestergaard et al. 1999). Genetic mapping has further revealed that the major quantitative trait locus responsible for the AD-like phenotype, designated derm1, is located on Chromosome 9, suggesting a strong genetic component to disease susceptibility in NC/Nga mice (Kohara et al. 2001). More recently, a stop-gain mutation in the *Clec10a* gene was identified as a critical factor enhancing hypersensitivity to house dust mite allergen. Restoration of *Clec10a* function ameliorated skin inflammation, highlighting a novel regulatory pathway that modulates TLR4-mediated immune responses in the skin (Kanemaru et al. 2019). Together, these findings demonstrate that NC/Nga mice offer a robust and genetically tractable model for dissecting the complex interplay of genetic, immunologic, and environmental factors in atopic dermatitis.

STR/OrtCrlj

The STR/Ort mouse is a well-established model of age-related, spontaneous primary osteoarthritis (OA). Initial studies revealed an inverse relationship between the expression of genes related to matrix remodeling and those related to lipid metabolism during OA progression, suggesting a fundamental role for metabolic regulation in disease development (Watters et al. 2007). Subsequent work showed that the earliest detectable lesions occur in the patellofemoral joint and may be caused by internal tibial torsion, highlighting joint geometry as a key factor in disease onset (Naruse et al. 2009). Gait analysis revealed that changes in paw area and treadmill task compliance correlate with OA onset in STR/Ort mice, allowing for noninvasive, longitudinal monitoring of disease progression independent of pain behavior. (Poulet et al., 2014). A recent comprehensive review emphasized that the STR/Ort mouse faithfully recapitulates many hallmarks of human primary OA, including cartilage degradation and subchondral bone remodeling (Staines et al., 2017). Genome-wide linkage analysis revealed that OA susceptibility is polygenic, with QTLs identified on chromosomes 4 and 5, underscoring the value of this model in exploring genetic mechanisms of OA (Watanabe et al., 2012). Collectively, these findings position the STR/Ort strain as a powerful resource for mechanistic studies of OA pathogenesis and for the development of novel therapeutic strategies.

JF1/Ms, and MSM/Ms, see Takada et al., 2022

References

Kanemaru K, Noguchi E, Tahara-Hanaoka S, Mizuno S, Tateno H, Fujisawa Y, Nakamura Y, Denda-Nagai K, Irimura T, Matsuda H et al. (2019) Clec10a regulates mite-induced dermatitis. Sci Immunol 4(42):eaax6908.
DOI: https://doi.org/10.1126/sciimmunol.aax6908

Kohara Y, Tanabe K, Matsuoka K, Kanda N, Matsuda H, Karasuyama H, Yonekawa H (2001) A major determinant quantitative-trait locus responsible for atopic dermatitis-like skin lesions in NC/Nga mice is located on chromosome 9. Immunogenetics 53:15–21.
DOI: https://doi.org/10.1007/s002510000286

Naruse K, Urabe K, Jiang SX, Uchida K, Kozai Y, Minehara H, Mikuni-Takagaki Y, Kashima I, Itomanet M (2009) Osteoarthritic changes of the patellofemoral joint in STR/OrtCrlj mice are the earliest detectable changes and may be caused by internal tibial torsion. Connect Tissue Res

50(4):243–255.
DOI: https://doi.org/10.1080/03008200902836065

Poulet B, de Souza R, Knights CB, Gentry C, Wilson AM, Bevan S, Chang YM, Pitsillides AA (2014) Modifications of gait as predictors of natural osteoarthritis progression in STR/Ort mice. Arthritis Rheumatol 66(7):1832–1842.
DOI: https://doi.org/10.1002/art.38616

Semba T, Nishimura M, Nishimura S, Ohara O, Ishige T, Ohno S, Nonaka K, Sogawa K, Satoh M, Sawai S et al. (2013) The FLS (Fatty liver Shionogi) mouse reveals local expressions of lipocalin-2, CXCL1 and CXCL9 in the liver with non-alcoholic steatohepatitis. BMC Gastroenterol 13:120. DOI: https://doi.org/10.1186/1471-230X-13-120

Soga M, Hashimoto S, Kishimoto Y, Hirasawa T, Makino S, Inagaki S (2010) Insulin resistance, steatohepatitis, and hepatocellular carcinoma in a new congenic strain of Fatty Liver Shionogi (FLS) mice with the Lepob gene. Exp Anim 59(4):407–419.
DOI: https://doi.org/10.1538/expanim.59.407

Soga M, Kishimoto Y, Kawaguchi J, Nakai Y, Kawamura Y, Inagaki S, Katoh K, Oohara T, Makino S, Oshima I (1999) The FLS mouse: a new inbred strain with spontaneous fatty liver. Lab Anim Sci 49(3):269–275.
PMID: 10403441

Staines KA, Poulet B, Wentworth DN, Pitsillides AA (2017) The STR/ort mouse model of spontaneous osteoarthritis – an update. Osteoarthritis Cartilage 25(6):802–808.
DOI: https://doi.org/10.1016/j.joca.2016.12.014

Suto H, Matsuda H, Mitsuishi K, Hira K, Uchida T, Unno T, Ogawa H, Ra C (1999) NC/Nga mice: a mouse model for atopic dermatitis. Int Arch Allergy Immunol 120(Suppl 1):70–75.
DOI: https://doi.org/10.1159/000053599

Takada T, Fukuta K, Usuda D, Kushida T, Kondo S, Kawamoto S, Yoshiki A., Obata Y, Fujiyama A, Toyoda A et al (2022) MoG+: a database of genomic variations across three mouse subspecies for biomedical research. Mamm Genome 33(1):31–43.
DOI: https://doi.org/10.1007/s00335-021-09933-w

Vestergaard C, Yoneyama H, Murai M, Nakamura K, Tamaki K, Terashima Y, Imai T, Yoshie O, Irimura T, Mizutani H et al. (1999) Overproduction of Th2-specific chemokines in NC/Nga mice exhibiting atopic dermatitis–like lesions. J Clin Invest 104(8):1097–1105.
DOI: https://doi.org/10.1172/JCI7613

Watanabe K, Oue Y, Miyamoto Y, Matsuura M, Mizuno Y, Ikegawa S (2012) Identification of a quantitative trait locus for spontaneous osteoarthritis in STR/ort mice. J Orthop Res 30(1):15–20. DOI: https://doi.org/10.1002/jor.21483

Watters JW, Cheng C, Pickarski M, Wesolowski GA, Zhuo Y, Hayami T, Wang W, Szumiloski J, Phillips RL, Duong LT (2007) Inverse relationship between matrix remodeling and lipid metabolism during osteoarthritis progression in the STR/ORT mouse. Arthritis Rheum 56(9):2999–3009.
DOI: https://doi.org/10.1002/art.22836
